# The Functional Roles and Applications of Immunoglobulins in Neurodegenerative Disease

**DOI:** 10.3390/ijms21155295

**Published:** 2020-07-26

**Authors:** Kyu-Young Sim, Kyeong Chan Im, Sung-Gyoo Park

**Affiliations:** School of Life Sciences, Gwangju Institute of Science and Technology (GIST), Gwangju 61005, Korea; rbdud0511@gist.ac.kr (K.-Y.S.); ikc412@gm.gist.ac.kr (K.C.I.)

**Keywords:** immunoglobulin, Alzheimer’s disease, Parkinson’s disease, diagnostic marker, immunotherapy

## Abstract

Natural autoantibodies, immunoglobulins (Igs) that target self-proteins, are common in the plasma of healthy individuals; some of the autoantibodies play pathogenic roles in systemic or tissue-specific autoimmune diseases, such as rheumatoid arthritis and systemic lupus erythematosus. Recently, the field of autoantibody-associated diseases has expanded to encompass neurodegenerative diseases such as Alzheimer’s disease (AD) and Parkinson’s disease (PD), with related studies examining the functions of Igs in the central nervous system (CNS). Recent evidence suggests that Igs have various effects in the CNS; these effects are associated with the prevention of neurodegeneration, as well as induction. Here, we summarize the functional roles of Igs with respect to neurodegenerative disease (AD and PD), focusing on the target antigens and effector cell types. In addition, we review the current knowledge about the roles of these antibodies as diagnostic markers and immunotherapies.

## 1. Introduction

Antibodies bind to various foreign antigens (e.g., bacterial components and products, viruses, protozoa, and fungi) that enter the circulatory system of both humankind and animals. However, some antibodies bind to self-molecules such as cellular components (including nucleic acids, phospholipids, and proteins) in healthy individuals; these are referred to as natural antibodies or autoantibodies. The majority of natural autoantibodies are immunoglobulin (Ig) M class; as such, they are polyreactive and bind several unrelated antigens with different affinities, thereby contributing to homeostasis of the immune system. However, the adaptive immune responses are mediated primarily by high-affinity, somatically mutated IgG antibodies [1,2]. As with B-2 cells, B-1 cells (which are the main cell type that produces IgM isotype natural autoantibodies) have a mechanism for somatic hypermutation and class-switching. Therefore, the ability to bind to self-antigens can be a template for emergence of high-affinity IgG antibodies that recognize self-antigens [2,3,4]. Therefore, IgG autoantibodies are also present in the plasma of healthy individuals; these IgG autoantibodies have personal specific signatures that tend to be stable over time [5]. Newborns share a universal immune profile with respect to the IgM repertoire; by contrast, IgG autoantibody repertories are highly diverse and shared between the mother and newborn. This suggests that IgG signatures change according to personal immune “experience” [6]. Naturally occurring autoantibodies may provide clues regarding disruption of immune homeostasis and autoimmune diseases associated with recognition of autoantigens. 

In some diseases, autoantibodies develop before clinical manifestations of autoimmune disease appear; examples include Sjögren’s syndrome (SS), autoimmune hepatitis, multiple sclerosis, systemic lupus erythematosus (SLE), rheumatoid arthritis (RA), and primary biliary cirrhosis. The roles of autoantibodies in the pathology of these diseases are different; indeed, these antibodies can have diverse effects against the same antigen depending on the target epitope [2,7,8,9,10,11]. Some autoantibodies in the sera of autoimmune disease patients exert various functions; for example, they act as pathogenic molecules that mimic hormone stimulation of receptors, block neural transmission by binding to receptors, affect signaling pathways, lyse cells, and induce inflammation at the site of autoantibody binding [8]. By contrast, some autoantibodies against autoantigens exposed during cell death increase phagocytosis of dead cells by forming a cell synapse between the phagocyte and the dead cell to induce engulfment [12]. Autoantibodies binding to the surfaces of necrotic cells from the serum of SLE patients increase phagocytic activity through complement component C4 [13]. Anti-dsDNA antibodies in sera from SLE patients participate in phagocytosis of the apoptotic cells by opsonizing the target cells [14]. Antiphospholipid antibodies also opsonize apoptotic cells by enhancing recognition of phagocytes [15]. In addition, apoptotic cell engulfment activates immunological signals that inhibit release of proinflammatory cytokines and induce an anti-inflammatory state in innate immune cells [12,16]. Therefore, autoantibodies can be used as biomarkers, thereby providing the opportunity to develop diagnostic tools and immunotherapies [2,7,8].

The concept of the brain as a site of immune privilege has been revised; we now know that immune cells provide immune surveillance within the central nerve system (CNS). Thus, research into the adaptive immune system is expanding into the CNS [17]. Mass cytometry of the mouse brain characterized the various resident and infiltrating immune populations in the brain compartment. The results indicate that small but significant numbers of immune cells, such as T cells, B cells, dendritic cells, and natural killer (NK) cells, migrate into the choroid plexus and meninges [18]. Lymphatic vessels in the brain have functional characteristics that include transportation of both fluid and immune cells; these vessels are connected to the deep cervical lymph nodes [19]. Therefore, many studies have examined the contributions of adaptive immune systems to neurodegenerative diseases such as Alzheimer’s disease (AD) and Parkinson’s disease (PD), which are related to neuroinflammation [20].

Recent clinical evidence shows that autoantibodies play roles in disease; therefore, the concept that neurodegenerative diseases may have an autoimmune etiology has been suggested [21,22,23]. Due to recent advances in technology related to antibody screening, many studies are attempting to identify autoantibodies associated with neurodegenerative diseases [24,25]. Some studies have identified autoantibodies that can either accelerate or prevent neurodegeneration, depending on the target antigens and cell types involved. Several reports show that Igs may be potential markers (on which we can base diagnostic techniques) and agents or targets for immunotherapy. In this review, we summarize the functional roles of Igs in the CNS, focusing on neurodegenerative diseases AD and PD, as well as their potential utility as diagnostic markers and immunotherapy agents or targets.

## 2. Role of Igs in AD

### 2.1. Alteration of Adaptive Immune Responses in AD 

AD is a degenerative disease of the brain, accounting for an estimated 60–80% of dementia cases. AD causes difficulties with memory, language, and problem-solving due to damage or destruction of nerve cells. The major hallmarks of AD are accumulation of beta-amyloid (Aβ) around neurons and tau phosphorylation inside neurons [26], which are accompanied by neurinflammation in the CNS [20]. A pathogenic form of Aβ, Aβ_1-42_, induces Aβ amyloid fibril formation; these accumulated Aβ amyloid fibrils in turn induce formation of senile plaques, resulting in neurotoxicity and induction of tau pathology. Tau is a microtubule-associated protein involved in microtubule polymerization and structural stabilization; however, the pathogenic form of tau forms aggregates and fibril seeds that damage the cell [27].

Recent research has focused on the contribution of the adaptive immune system to AD pathogenesis. One study shows that the number of clonally expanded CD8^+^ T effector memory CD45RA^+^ cells in the blood of AD patients is increased, and that these cells patrol the cerebrospinal fluid (CFS) [28]. Lymphocyte profiling has revealed a significant decline in CD4^+^ T cell populations in the CSF in brain regions in which Aβ is deposited. By contrast, the Aβ burden shows a positive correlation with increased numbers of memory B cells in the CSF; this is exacerbated in *APOE ε4* carriers [29]. Increased numbers of double-negative (IgD^−^CD27^−^) memory B cells and a reduction in the number of naïve B cells (IgD^+^CD27^−^) in the peripheral blood have been identified in AD [30], and the number of cells producing antibodies targeting Aβ_1-42_ is increased in AD [31]. These studies suggest a functional role for the adaptive immune system in AD pathogenesis, particularly with respect to B cell function [32].

### 2.2. Evidence for an Association between Ig Responses and AD

Clinical evidence suggests that the Ig response is associated with AD pathogenesis. Ig labeling shows that Ig^+^ neurons have neurodegenerative apoptotic characteristics that are not observed in Ig^−^ neurons [33]; Ig binding to apoptotic debris may induce apoptotic cell clearance [12,14,15]. Additionally, C1q and C5b-9 were detected in these Ig^−^ positive neurons [22]. In the brain of human AD patients immunized (or not) with AN-1792, IgG was detected in Aβ plaques, on plaques surrounding microglia, and within neurons adjacent to plaques; antibody deposition correlated with the C1q load [34]. Biochemical studies provide evidence for an interaction between IgG and tau protein, supporting a pathological role for Igs in AD brains [35]. These data support the autoimmune disease theory for AD; blood–brain barrier (BBB) dysfunction in AD allows autoantibodies access to targets in the brain, which leads to autoimmunity-induced neural cell death [22]. In addition, changes of autoantibody levels in blood and CSF have been identified. The blood of AD patients contains higher levels of Aβ-IgG immune complexes than that of controls. In addition, the levels of Aβ-IgG complexes correlate negatively with performance in cognitive tests [36]. Antibodies specific for tau protein and heavy neurofilaments are increased in AD patients [37]. Moreover, elevated plasma levels of nicotinic acetylcholine receptor α7 (α7 nAChR)-specific autoantibodies are characteristic of early AD patients [38]. The neuronal α7 nAChRs are widely distributed in the CNS and are involved in PI3K/Akt signaling, which protects brain cells from apoptotic signals [39,40]. The α7 nAChRs interact directly with Aβ peptide and stimulate the neuroprotective pathways that protect against Aβ-toxicity [41]. Studies in an lipopolysaccharide injected mice model show that α7 nAChRs-specific antibodies aggravate neuroinflammation by stimulating proinflammatory cytokines and downregulating anti-pro-inflammatory miRNAs, which are responsible for limiting inflammatory signals in the CNS [42]. An autoantibody screening study identified autoantibodies in CSF that target glia-derived nexin, actin-interacting proteins, metalloproteinase inhibitor 2, quinone oxidoreductase, inositol trisphosphate receptor 1, and endoplasmic reticulum calcium ATPase 2 [43]. The levels of autoantibodies targeting ATP synthase, angiotensin II type 1, and 5-hydroxytryptamine also are altered in AD [44]. The concentration of IgM and IgG autoantibodies targeting alpha B-crystallin is increased in AD patients [45]. These data suggest that some types of Ig have functional roles related to AD pathogenesis.

### 2.3. BBB Breakdown and Ig Infiltration of the CNS

The brain has a lymphatic drainage system for clearing waste, which includes abnormal proteins that contribute to neurodegeneration [46,47]; in addition, meningeal lymphatics vessels provide a direct pathway by which CSF components and immune cells drain to the cervical lymph nodes [48]. Therefore, neuronal antigens could be presented to immune cells in cervical lymph nodes. However, as antineuronal surface antibodies are rarely found in the CSF and blood of healthy participants, it seems that production of neuronal surface antibodies is induced by neuronal inflammatory conditions [49].

Some evidence suggests that Igs in the CNS have infiltrated from the blood. A study of CSF and blood from hip fracture patients showed that CSF and blood contain similar autoantibody repertoires [50]. However, the amounts of each antibody type in the CSF were far lower than those in blood [50,51]. The similarity of the autoantibody repertoires and the lower amounts of antibodies in the CSF suggest that blood Igs infiltrate the CNS. Additionally, low levels of IgG were observed in the adult human cortex; in particular, diffuse patterns were observed around blood vessels, but extending into the brain parenchyma [52].

BBB breakdown may result in greater infiltration of the brain by Igs. BBB breakdown was identified in AD post-mortem human studies measuring perivascular accumulation of blood-derived fibrinogen, Ig, thrombin, albumin, and loss of BBB tight junctions. In addition, genetic risk factors for AD, such as *APOE*, *APP*, and *PSEN1*, induce BBB breakdown in animal models [53,54,55,56]. A study revealed that physical BBB breakdown induced by needle insertion can trigger selective IgG localization in the brain parenchyma at the site of injury; in addition, IgG antibodies from neuromyelitis optica spectrum disease patients induce astrocyte pathology in this region [57]. These studies suggest that BBB breakdown in AD results in deposition of Igs in the brain. In addition, failure of the lysosome system in AD could be another possible factor that causes antibody deposition in the brain [58,59]. 

### 2.4. Protective Role of Natural Antibodies in AD

Many studies have identified functional roles for Igs in AD. However, the function of Igs depends on the target antigen and CNS cell type. Some reports suggest a protective role for IgG in AD. Studies identified an increase in IgG associated with microglia in the brains of AD transgenic mice [60,61]. One study used the immune-deficient AD mouse model, which lacks B, T and NK cells, to examine the functional role of Igs. They noted several changes in the immune-deficient AD mouse model, including increased levels of Aβ and several proinflammatory cytokines, and a shift in microglial phenotype. Delivery of normal mouse IgG to mice via either bone marrow transplantation or direct stereotactic injection led to a marked reduction in AD pathology. The Src/spleen tyrosine kinase (Syk)/phosphatidylinositol 3-kinase signal transduction pathway is involved in the IgG-induced uptake of Aβ by microglia [60]. Another study showed that stress granules (SGs) containing Syk and phosphotyrosine are prevalent in the brains of AD patients. These SGs modulate Syk and cause microglial cell dysfunction with respect to phagocytosis of *Escherichia coli* or Aβ fibrils. Phagocytic activity was restored by treatment with non-specific rabbit IgG, suggesting a mechanism that explains the therapeutic efficacy of intravenous IgG [62]. Another research study examined how AD risk factors (*APOE* genotype, aging, and gender) affect IgG levels in various brain regions, especially in AD-susceptible regions, such as the hippocampus and cortex. IgG was detected mainly on microglia and some neurons, but not astrocytes [61]. These data show that Igs might prevent Aβ pathology by increasing phagocytosis by microglial cells, leading to increased clearance of Aβ. Another study showed that human IgG prevents Aβ-mediated neurotoxicity by inhibiting Aβ aggregation. The protective effects of IgG seem to be independent of IgG specificity; the Fab region is responsible for inhibiting Aβ aggregation, and bioinformatics tools show that the Fab regions bind to Aβ [63]. These studies show that non-specific IgG may also inhibit Aβ aggregation. 

Antibodies specific for some epitopes are protective against AD. Natural antibodies specific for toxic Aβ and amyloidogenic non-Aβ species are present in blood and CSF samples from both AD patients and healthy controls. IgG targeting oligomeric Aβ_1-42_ declines with age and advancing AD, and IgG isolated from the plasma of AD patients or healthy controls protects primary neurons from Aβ-induced toxicity [51]. These results suggest that natural Igs, even in healthy individuals, protect against AD pathogenesis. Thus, administering these antibodies to the elderly may prevent AD (Table 1).

### 2.5. Pathogenic Natural Antibodies in AD

By contrast, some research shows that Igs have pathogenic effects. The Fc gamma receptor (FcγR) is a receptor for IgG that has both activating and inhibitory activities [75,76]. Multiple cell types within the CNS (e.g., neurons and astrocytes) express FcγRs, and various stimuli (e.g., immune complexes) can activate expression of FcγRs [67,76,77,78]. FcγRs participate in uptake of Igs and increase calcium concentrations and neurotransmitter release from motor neurons [79]. IgG immune complexes increase phosphorylation of extracellular signal-regulated kinase and levels of intracellular calcium, and induce FcγR-mediated internalization of IgG in cortical and hippocampal cells [77]. In addition, IgG immune complexes activate sensory neurons directly [68]. These findings suggest that IgG immune complexes affect neuronal function via direct interactions with FcγR expressed by non-immune cells in the brain.

Increased expression of FcγRs in human brain tissue from individuals with neurodegenerative diseases, such as AD and PD, along with ligation of specific FcγRs in the CNS by IgG immune complexes and alternate ligands, promotes neuroinflammation and increases neurodegeneration [67,69,78]. For example, neuronal cells exposed to synthetic Aβ and the hippocampus of AD brains both show upregulation of FcγRIIb, which activates endoplasmic reticulum stress and caspase-12; knockout of FcγRIIb induces resistance to synthetic Aβ-induced cell death in vitro. Moreover, genetic depletion of FcγRIIb rescued memory impairment in an AD mouse model [80]. A study using the *APOE*-deficient mouse showed upregulation of Fc receptors, predominantly type IV, and increased amounts of serum and brain IgGs compared with controls. Interestingly, FcγR engagement induces Aβ accumulation in neurons, but genetic deletion of the γ-chain of activating Fc receptors reduces learning and memory impairment. In vitro tests show that activation of Fc receptors increases expression of mitogen-activated protein kinases and β-site amyloid precursor protein cleaving enzymes, which cause hyperphosphorylation of tau, Aβ accumulation, and synapse loss in primary neurons [81]. This suggests that immune complexes induce FcγR overexpression by neurons and that activation of neuronal FcγRs induces AD-like pathology. Some antibodies can have pathogenic effects in AD, depending on the target antigen. AD model mice show age-dependent increases in expression of antibodies targeting ceramide, suggesting that autoimmune reactions against ceramide play a role in AD pathogenesis. In addition, induction of serum anticeramide IgG by ceramide administration increases the plaque burden in female mice compared with controls, suggesting that systemic anticeramide IgG and exosome levels correlate with increased plaque formation [70].

Activation of microglia also has functional effects on neurodegeneration. Activation of microglia increases phagocytosis, clearance, and degradation of Aβ, which prevents formation of Aβ plaques in the brain. However, prolonged activation of microglia leads to release of proinflammatory cytokines, which initiate a proinflammatory cascade [71]. Immune complexes play important roles in IgG-mediated phagocytosis by microglia, and these cells are also affected by cytokines in the CNS [82]. However, immune complexes also induce inflammatory reactions in the brain parenchyma and induce neuronal tissue damage indirectly through activation of FcγR on microglia [69]. IgG in lupus serum induces M1 polarization of brain microglia. IgG antibodies bind to microglia through the Fc region; then, the B cell activating factor in lupus serum upregulates expression of FcγR on the surface of microglia, thereby triggering FcγR-mediated signaling pathways to induce inflammatory responses [83]. Another study revealed that tau antibodies (both with and without full effector function) in the presence of microglia protect neurons from tau toxicity, but effector tau antibodies induce release of proinflammatory cytokines by microglia [64].

**Figure 1:**
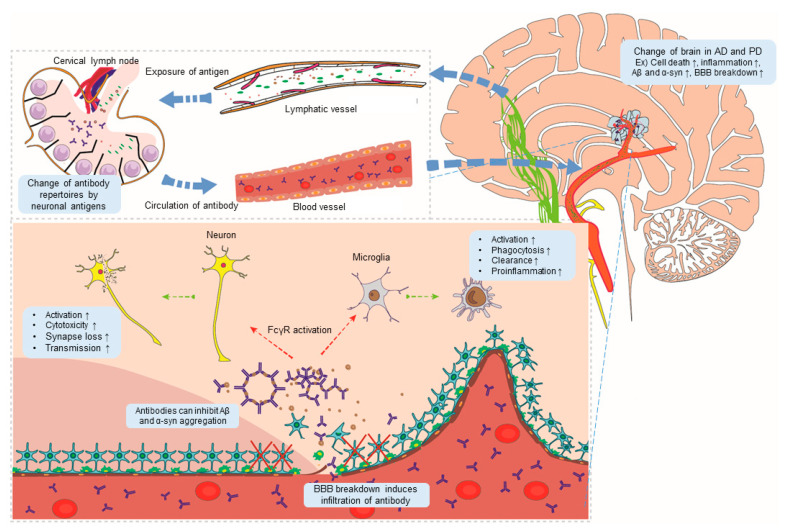
A model showing antibody-mediated pathogenesis of Alzheimer’s disease (AD) and Parkinson’s disease (PD). Changes in brain conditions in AD and PD (for example, cell death, inflammation, and aggregation of abnormal proteins) promote the outflow of neo-antigens through lymphatic vessels, causing changes in antibody repertoires. Damage to the blood–brain barrier (BBB) generated in AD and PD induces infiltration by antibodies. Antibodies activate FcR expression by brain cells, thereby promoting pathogenesis of AD and PD in the brain. Aβ: beta-amyloid; α-syn: α-synuclein.

IgG from AD and PD are more toxic to neurons than control IgG [72,84]. In addition, disease alters IgG Fc sugar moieties, known as *N*-glycans, which affect the affinity of the Fc domain [85,86]. Therefore, antibody function is complex; in particular, function can differ depending on enviromental conditions, effector cell type, and target antigen (Table 1). Therefore, it is necessary to comprehensively consider various factors, including FcγR-IgG ligation, when developing treatment modalities based on antibodies. Some studies show that anti-Aβ and -tau antibodies with no effector function can inhibit AD pathogenesis, suggesting utilization of immunotherapies based on modified antibodies that lack adverse effects [64,87,88]. Figure 1 summarizes a model of antibody-mediated pathogenesis in neurodegenerative disease.

### 2.6. Diagnostic Application of Antibodies for AD

As the number of people developing AD is expected to increase, we need to develop low-cost and patient-friendly diagnostic techniques based on blood biomarkers. Autoantibodies are one candidate blood biomarker; indeed, anti-Aβ and -tau protein antibodies have been used as biomarkers [44]. Autoantibodies targeting Aβ have been studied by many groups. A meta-analysis of 2901 individuals (1311 AD patients and 1590 healthy control subjects) revealed a significant increase in the amount of endogenous IgG autoantibodies targeting Aβ in the blood; by contrast, anti-Aβ IgM autoantibody levels were markedly lower in patients with AD than in control subjects, suggesting the possibility that alterations in autoantibody levels may be a biomarker [89]. With respect to anti-tau antibodies, several groups have measured concentrations in the blood. However, there were no statistically significant differences between healthy controls and AD patients [90]. This lack of difference was also shown in another study [91].

Various technological advances related to high-throughput antibody screening have meant that many studies are attempting to identify other antibodies that may be blood biomarkers for AD. Microarrays for screening of autoantibodies have identified a combination of autoantibodies in blood that can differentiate patients with mild cognitive impairment from age-matched controls with high accuracy. The target proteins are a variety of receptors, adapters, kinases, cytoskeletal components, potassium channel subunits, ribosomal and mitochondrial proteins, and other accessory proteins [25]. Furthermore, 44 autoantibodies from the panel enrolled in a previous study can distinguish prodromal AD pre-surgically and with high accuracy in individuals admitted to the hospital for hip fracture repair surgery [92]. In addition, various protein and peptide arrays have led to identification of autoantibodies specific to patients with AD [4,93]. These antibodies target proteins involved in synaptic activity, including neurotransmitters [94,95] and receptors [96], and autoantibodies specific for proteins modulating inflammation [95] and the BBB [96]. The levels of autoantibodies targeting proteins involved in energy metabolism, such as aldolase and adenosine triphosphate synthase β, are higher in the serum of patients with AD than in controls [93]. This suggests that the antibody repertoire responsible for AD pathogenesis changes; such changes could be utilized as blood biomarkers for AD. However, actual application would require clear criteria for calculating clinical scores on which a diagnosis of AD would be based.

### 2.7. Therapeutic Application of Antibodies in AD

Several immunotherapies have been developed using antibodies against Aβ, the major marker of AD. Two immunotherapeutic strategies, active immunization and passive immunization, have been used as immune therapies [76]. Passive immunotherapy with antibodies against Aβ is based on binding of transferred IgG to Aβ, which may prevent or inhibit AD pathogenesis. However, the lack of clinical benefit has led to repeated failures of clinical trials. Phase 3 clinical trials (NCT01900665) revealed that solanezumab, a monoclonal antibody against Aβ, showed no significant benefit with respect to preventing cognitive decline in patients with mild AD [97]; this failure suggests that earlier treatment may be critical for cognitive benefits. Phase III trials (NCT00667810 and NCT00676143) also confirmed that bapineuzumab (as a passive vaccination for mild to moderate AD) lacks efficacy [98]. A study of gantenerumab was halted early due to lack of a positive effect (NCT01224106) [99], but high doses of gantenerumab did result in robust Aβ plaque removal (NCT01224106 and NCT02051608) [100]. An active vaccine against Aβ was also used to stimulate antibody production. One of these, AN-1792, comprises synthetic full-length Aβ peptide plus a QS-21 adjuvant. Long-term follow-up patients immunized with AN1792 revealed sustained anti-AN1792 antibody titers and a marked reduction in functional decline and plaque numbers [101,102]. However, the vaccination caused meningoencephalitis in some cases [103]. Nevertheless, to date, researchers are developing and testing several possible interventions in clinical trials; these are based on anti-Aβ, anti-tau antibodies, and agents that prevent neuroinflammation (Table 2) [76,104].

## 3. Role of Igs in PD

### 3.1. Changes to the Adaptive Immune System in PD

PD is a progressive neurodegenerative disease that affects 2–3% of the population aged ≥65 years. PD results in impaired movement and cognitive problems, which become increasingly prevalent as the disorder progresses. The major hallmarks of PD are accumulation and aggregation of α-synuclein (α-syn) and loss of neurons in the substantia nigra (SN) [105]. Although neuroinflammation may not be the first step in PD, neuroinflammation is strongly implicated in disease pathogenesis [20]. Therefore, several studies have profiled the lymphocyte populations in PD to identify the role of the adaptive immune system. The data show that in human and mouse models of PD, there are fewer naïve T cells, CD4^+^ Th (helper) cells, and Treg cells than in controls. Also, there is an increasing tendency toward clonally expanded CD45RA^+^ cells, activated microglial cells, and activated T cells in the CSF of patients with PD [28,106,107]. Another study reports that the CD4^+^ T cell population in PD has a Th1-biased profile [108]. However, there is a lack of evidence related to changes in the B cell population in PD [107]. 

### 3.2. Evidence for an Association Between Ig Response and PD

PD is associated with altered Ig levels. One study identified an increased CSF/serum IgG ratio in cases of advanced PD [109]. Another showed that the subthalamic nucleus of the brain in PD patients showed widespread extravascular staining for IgG, indicating BBB impairment; however, IgG staining was restricted to the lumen of vessels in the brains of healthy controls [110]. Another study revealed that some pigmented dopamine neurons in the SN of PD patients have more IgG than those of healthy controls, and that the IgG co-localizes with α-syn. IgG-positive neurons correlate negatively with the degree of cell loss and positively with the number of HLA-positive microglia. The most common subclass of neuronal IgG in the damaged SN is IgG1; in addition, the high-affinity activating IgG receptor, FcγRI, is expressed on nearby activated microglia [111]. These results suggest that Igs play functional roles related to activation of microglia, leading to loss of dopamine nigral neurons in PD. 

The majority of studies of Igs have focused on changes in the amounts of α-syn antibodies, but the results are inconsistent and contradictory [112,113]. However, a study that measured the affinity of these antibodies revealed that the amount of high-affinity α-syn antibodies in the plasma of PD patients is lower than that in healthy controls, and that there are markedly fewer α-syn–antibody immunocomplexes in the plasma of PD patients. Furthermore, cross binding of α-syn antibodies to β- and γ-synuclein monomers suggests that antibodies target mainly C-terminal epitopes [114]. Another group showed that glycosylation of IgG was significantly different between patients with PD and controls. They suggest that the type of IgG glycosylations in PD decrease its capacity to inhibit Fcγ-RIIIα binding, so IgG may trigger antibody-dependent cell cytotoxicity and inflammation in individuals with PD [86].

### 3.3. BBB Breakdown in PD

Similar to AD, studies report BBB breakdown in PD, which may allow antibody infiltration. One study used positron emission tomography to show BBB dysfunction in the midbrain of PD patients relative to controls [115]. Cerebral microbleeds are more common in PD patients with dementia than in those without, suggesting that the burden of cerebral microbleeds may contribute to further cognitive impairment in PD [116]. In addition, studies show markedly increased permeability of the BBB in the post-commissural putamen of PD patients [117], as well as microvascular changes in post-mortem SN samples [110]. There are high levels of αvβ3 expression in the locus ceruleus and the SN pars compacta of PD brains, and the presence of αvβ3 reactive vessels suggests newly created vessels that have not developed restrictive properties of the BBB [118]. This evidence suggests that the brain of PD patients is susceptible to infiltration by antibodies, as in AD.

### 3.4. Protective Role of Natural Antibodies in PD

Igs play a bifunctional role (protecting or accelerating) in PD pathogenesis. In particular, antibodies against α-syn protect against PD. One of the antibodies secreted by memory B cells in PD patients has high affinity for epitopes at the C terminus of α-syn. An in vitro α-syn seeding assay revealed that these antibodies neutralize the seeding of intracellular syn aggregates [65]. Immunization against α-syn results in reduced α-syn accumulation and synaptic loss in mouse models of Lewy body disease [66,119,120]. Antibodies against α-syn induce clearance of extracellular α-syn proteins by microglia through FcγR binding. In addition, stereotaxic administration of antibodies into the brain prevent neuron-to-astroglia transmission of α-syn, thereby inducing increased co-localization of α-syn and the antibody on microglia [66]. These findings suggest that antibodies against α-syn play protective roles and can be utilized as potential therapeutic targets (Table 1).

### 3.5. Pathogenic Role of Natural Antibodies in PD

Igs also play a role in disease pathology, principally through FcγR activation. Activation of FcγR expression by neurons induces PD pathogenesis as follows: α-syn fibrils bind to FcγRIIB of neurons; FcγRIIB then mediates cell-to-cell transmission of α-syn via activation of Src homology region 2 domain containing phosphatase-1/2. Blocking this signaling pathway attenuates formation of Lewy-body-like inclusion bodies [74]. FcγRs are expressed on the plasma membrane of microglia; these receptors bind IgG and activate microglia, resulting in damage to dopaminergic neurons in the SN. Virus-induced overexpression of α-syn in wild-type animals induces activation of NF-κB p65 and other proinflammatory molecules, which activate microglia and result in loss of DA neurons from the SN. However, FcγR^−/−^ mice exhibit no neuritic changes in the absence of α-syn-induced neurodegeneration; these mice show attenuated microglial activation [73]. In vitro experiments show that IgG in PD activates microglia via the FcγR to induce dopaminergic cell injury. Stereotaxic injection of PD IgG into the SN of mice increases the number of microglial cells, accompanied by a 40% loss in tyrosine hydroxylase (TH)-positive neuronal cells in the SN. However, there is no significant increase in the number of microglia and no loss of TH-positive cells in FcγR^−/^^−^ mice [121]. In PD patients, antimyelin-associated glycoprotein (MAG)–IgM autoantibody levels are significantly higher than those in healthy control or patients with atypical parkinsonism. Furthermore, studies report increasing levels of antimyelin basic protein and antiproteolipid protein IgM autoantibodies in PD [122,123]. The MAG–IgM autoantibodies induce neuropathy, which is a common symptom of PD [124,125,126]. IgG from PD and recombinant human C5a act synergistically to induce selective dopaminergic neurodegeneration in rat mesencephalic neuron–glia cultures; however, IgG from disease controls and normal controls does not show dopaminergic neurotoxicity. The results from microglia-supplemented neuronal cultures indicate that microglia play pivotal roles in neurotoxicity. PD IgG and C5a act synergistically to activate microglia, which produce superoxide and nitric oxide, both of which are neurotoxic. Finally, F(ab′)_2_ fragments of PD IgG play important roles in neurotoxicity [72]. Taken together, these results suggest that FcγR-IgG interactions, especially IgG from PD patients, activate microglia, resulting in loss of neurons and activated synuclein transmission (Table 1 and Figure 1). 

### 3.6. Diagnostic Application of Antibodies in PD

Identifying blood biomarkers for PD is the goal of much research, however an effective biomarker remains elusive. However, antibodies in the blood of PD patients are promising candidate PD biomarkers. In particular, α-syn IgG is a strong candidate. A study of four IgG subclasses (IgG1, IgG2, IgG3, and IgG4) found higher levels of anti-α-syn IgG2 and lower levels of anti-α-syn IgG4 in PD than in controls. Anti-α-syn IgM levels were also lower in PD. Increased or decreased levels of IgG subclasses are expected to be one biomarker of PD [112]. A recent study reports that the repertoire of high-affinity or -avidity IgG autoantibodies targeting a-syn is significantly reduced in PD [127]. The pathological changes associated with PD might contribute to the levels of naturally occurring α-syn autoantibodies in both serum and CSF. Both of these are potential biomarkers for PD patients [128,129]. However, studies of α-syn antibodies report inconsistent measurements of antibody levels. Several studies demonstrated that serum α-syn antibody titers in patients are different depending on age, disease duration, severity, and genetic inheritance. The distinct pattern of pathology and symptom progression associated with some isolated naturally occurring human anti-α-syn antibodies might be a potent diagnostic marker for PD [65,112,113]. However, a systematic review and meta-analysis highlight many caveats to this conclusion based on the limitations of the assays used, the clinical heterogeneity of the study cohorts, the lack of longitudinal data, and poor matching of controls to patients; thus, the overall quality of the evidence is poor. Hence, the value of α-syn autoantibodies as diagnostic or prognostic biomarkers remains uncertain [113]. Another study tried to identify changes in a panel of autoantibodies in the blood of PD. They selected a panel of blood-borne autoantibodies as biomarkers, which distinguished early-stage PD subjects from controls with an overall accuracy of 87.9%; it also distinguished PD from neurological diseases. These autoantibodies target microtubule-affinity-regulating kinase 1, pseudouridine synthase-like 1, interleukin 20, and C-C motif chemokine ligand 19, among others [24].

### 3.7. Therapeutic Application of Antibodies in PD

Since α-syn is a major marker for PD pathogenesis, use of α-syn antibodies has been put forward as a potential immunotherapy. Anti-α-syn antibodies may stimulate microglial cells to scavenge extracellular α-syn and prevent its transfer from one neuron to another. Current immunotherapies for PD target microglia activation, which regulates lysosome function for effective clearance from the brain [130]. A passive vaccination for PD, prasinezumab (PRX002; a humanized IgG1 monoclonal antibody), has been developed. Clinical phase 1 (NCT02157714) trials show that the antibody can regulate serum α-syn levels in a dose-dependent manner [131]; thus, passive vaccination for PD is being examined in a clinical phase 2 trial (NCT03100149). Active vaccination with a synthetic α-syn-mimicking peptide called Affitope (PD01A) resulted in a significant increase in PD01-specific antibody titers; these antibodies bind to both oligomeric and fibrillar α-syn. At week 26, there was a trend toward a reduction in the levels of oligomeric α-syn in plasma and CSF after treatment with PD01A. Therefore, PD01A is being prepared for a phase 2 clinical trial. A second vaccination with PD03A resulted in a clear dose-dependent immune response against the α-syn-targeted epitope over time, with antibody reactivation upon booster immunization (NCT02267434); however, a parallel phase 1 study did not find any immune responses (NCT02270489) [132,133]. Multiple immunotherapeutic agents for PD are currently in clinical trials (Table 2) [134]. 

## 4. Conclusions

The increased or decreased risk of AD and PD due to autoimmune mechanisms suggests that autoantibodies produced in those with autoimmune diseases can affect neurodegeneration. A previous study showed that primary SS patients have a 2.69-fold increased risk of developing AD; the risk is even higher in older people [135]. Another study found that 18 out of 25 autoimmune diseases were significantly associated with a risk of dementia [136]. Patients with autoimmune rheumatic diseases such as RA and SS have a significantly higher risk of PD [137]. By contrast, patients of SLE have a lower risk of PD [138]. These results suggest that autoimmune disease, possibly through autoantibodies, can either induce or prevent neurodegenerative conditions. However, further research is needed to identify the specific autoantibodies that are associated with neurodegeneration and to identify their functions.

In recent decades, extensive efforts by researchers, together with advances in molecular and biochemical techniques, have made it possible to elucidate the lymphatic drainage system in the brain and examine infiltrating adaptive immune components in the CNS. In particular, the appreciation of the relationship between Igs and neurodegenerative disease such as AD and PD and the potential role of Igs in disease pathology have grown markedly. Our summary indicates that the functional roles of Igs depend on effector cells and target antigens. Igs or immune complexes can be neurotoxic by inducing proinflammatory responses through microglia activation in the CNS via activation of FcγR. By contrast, they may exert protective effects through triggering immune-complex-mediated phagocytosis by microglia via FcγR activation. Interestingly, IgG from PD and AD patients are more neurotoxic than those from controls, suggesting that regulation of Ig-mediated FcγR signaling is a complicated process controlled by additional factors. Recently, studies have shown that Ig glycosylation affects the affinity of antibodies for FcγR. In addition, microarray studies identified promising autoantibodies in the blood of AD and PD patients. Data suggest that changes in both Fab and Fc regions of Igs have important roles with respect to interactions with CNS cells in neuronal disease. Although many attempts have been made to develop vaccines based on antibodies specific for Aβ and α-syn, most have failed. A more comprehensive understanding of the function and roles of Fc and Fab fragments may enable us to develop advanced vaccines for the treatment of CNS disease.

## Figures and Tables

**Table 1:** The potential roles of autoantibodies in neuronal degenerative diseases.

**Protective Role**				
**Disease**	**Function**	**Antibody Target**	**Effector Cell**	**Reference**
AD	Enhance the phagocytosis of Aβ	Non-specific	Microglia	[60,61,62]
	Inhibit Aβ aggregation	Fab-mediated function	Cortical Neuron Endothelial cell	[63]
	Neutralize Aβ-induced toxicity	Oligomeric Aβ_1-42_	Primary Neuron	[51]
	Reduce tau toxicity	Tau	Microglia	[64]
PD	Neutralize the intracellular α-syn aggregation	α-syn	Neuron	[65]
	Remove the extracellular α-syn proteins		Microglia	[66]
	Prevent α-syn transfer between neuron and astrocyte			
**Pathogenic Role**				
**Disease**	**Function**	**Antibody Target**	**Effector Cell**	**Reference**
AD	Induce the neuroinflammation as Immune complexes	FcγR-mediated function	FcγR-expressing cells in the CNS	[67,68,69]
	Increase Aβ plaque formation	Ceramide	Neuron Endothelial cell	[70]
	Release the proinflammatory cytokines	FcγR-mediated function	Microglia	[71]
	Release the proinflammatory cytokines	Tau		[64]
PD	Induce neuronal damage via microglial activation	FcγR-mediated function	Microglia	[72,73]
	Promote the α-syn transfer		Neuron	[74]

AD: Alzheimer’s disease; Aβ: beta-amyloid; PD: Parkinson’s disease; α-syn: α-synuclein; FcγR: Fc gamma receptor.

**Table 2:** Clinical trials of immunotherapies for neuronal degenerative diseases.

Drug	Antibody Type (Target)	FDA Status	NCT (Patients)
Solanezumab	Passive (Mid-domain of the Aβ peptide)	Phase 3	NCT02008357 (Older individuals who may be at risk for memory loss) NCT02760602 (Prodromal AD)
Bapineuzumab	Passive (*N*-terminal region of Aβ)	Discontinued	NA
Gantenerumab	Passive (Conformational epitope on Aβ fibrils)	Phase 3	NCT03443973 and NCT03444870 (Early AD)
Crenezumab	Passive (Multiple forms of aggregated Aβ)	Phase 2	NCT02670083 (Prodromal to mild AD) NCT01998841 (Autosomal-dominant AD) NCT01397578 (Mild to moderate AD)
Aducanumab	Passive (Conformational epitope on Aβ)	Phase 3	NCT02477800 and NCT02484547 (Early AD)
BAN2401	Passive (Large, soluble Aβ protofibrils)	Phase 3	NCT01767311 and NCT03887455 (Early AD)
AN-1792	Active (Synthetic full-length Aβ peptide with QS-21 adjuvant)	Discontinued	NA
Gosuranemab	Passive (*N*-terminal fragments of tau)	Phase 2	NCT03352557 (Early AD)
AADvac1	Active (Synthetic tau peptide withaluminum hydroxide adjuvant)	Phase 2	NCT01117818 (Early AD)
Prasinezumab	Passive (Against aggregated α-syn)	Phase 2	NCT02157714 and NCT03100149 (PD)
BIIB054	Passive (α-syn residues 1-10)	Phase 2	NCT03318523 and NCT02459886 (PD)
ABBV-0805	Passive (Oligomeric/protofibrillar α-syn)	Phase1	NCT04127695 (PD)
Activa Affitope (PD01A)	Active (Synthetic α-syn peptide with adjuvant)	Phase1	NCT02618941 (PD)

Aβ: beta-amyloid; AD: Alzheimer’s disease; α-syn: α-synuclein; PD: Parkinson’s disease.

## Abbreviations

Ig	Immunoglobulin
SS	Sjögren’s syndrome
SLE	Systemic lupus erythematosus
RA	Rheumatoid arthritis
AD	Alzheimer’s disease
PD	Parkinson’s disease
CNS	Central nervous system
CSF	Cerebrospinal fluid
NK	Natural killer
BBB	Blood–brain barrier
α7 nAChR	Nicotinic acetylcholine receptor α7
Syk	Spleen tyrosine kinase
FcγR	Fc gamma receptor
SGs	Stress granules
Aβ	Beta-amyloid
SN	Substantia nigra
α-syn	α-Synuclein
TH	Tyrosine hydroxylase
MAG	Myelin-associated glycoprotein
